# Integrating evolutionary theory and social–ecological systems research to address the sustainability challenges of the Anthropocene

**DOI:** 10.1098/rstb.2022.0262

**Published:** 2024-01-01

**Authors:** Thomas E. Currie, Monique Borgerhoff Mulder, Laurel Fogarty, Maja Schlüter, Carl Folke, L. Jamila Haider, Guido Caniglia, Alessandro Tavoni, Raf E. V. Jansen, Peter Søgaard Jørgensen, Timothy M. Waring

**Affiliations:** ^1^ Human Behaviour and Cultural Evolution Group, Centre for Ecology and Conservation, University of Exeter, Penryn Campus, Penryn TR10 9FE, UK; ^2^ Department of Anthropology, University of California Davis, Davis, CA 95616, USA; ^3^ Santa Fe Institute, Santa Fe, NM 87506, USA; ^4^ Max-Planck Institute for Evolutionary Anthropology, 04103 Leipzig, Germany; ^5^ Stockholm Resilience Centre, Stockholm University, SE-106 91 Stockholm, Sweden; ^6^ Beijer Institute of Ecological Economics, The Royal Swedish Academy of Sciences, SE-104 05 Stockholm, Sweden; ^7^ Konrad Lorenz Institute for Evolution and Cognition Research, A-3400 Klosterneuburg, Austria; ^8^ Department of Economics, University of Bologna, 40126 Bologna, Italy; ^9^ Grantham Research Institute on Climate Change and the Environment, London School of Economics, London WC2A 2AE, UK; ^10^ Global Economic Dynamics and the Biosphere, Royal Swedish Academy of Sciences, SE-104 05 Stockholm, Sweden; ^11^ Mitchell Center for Sustainability Solutions and School of Economics, University of Maine, Orono, ME 04469-5710, USA

**Keywords:** evolution, theory, social-ecological systems, Anthropocene

## Abstract

The rapid, human-induced changes in the Earth system during the Anthropocene present humanity with critical sustainability challenges. Social–ecological systems (SES) research provides multiple approaches for understanding the complex interactions between humans, social systems, and environments and how we might direct them towards healthier and more resilient futures. However, general theories of SES change have yet to be fully developed. Formal evolutionary theory has been applied as a dynamic theory of change of complex phenomena in biology and the social sciences, but rarely in SES research. In this paper, we explore the connections between both fields, hoping to foster collaboration. After sketching out the distinct intellectual traditions of SES research and evolutionary theory, we map some of their terminological and theoretical connections. We then provide examples of how evolutionary theory might be incorporated into SES research through the use of systems mapping to identify evolutionary processes in SES, the application of concepts from evolutionary developmental biology to understand the connections between systems changes and evolutionary changes, and how evolutionary thinking may help design interventions for beneficial change. Integrating evolutionary theory and SES research can lead to a better understanding of SES changes and positive interventions for a more sustainable Anthropocene.

This article is part of the theme issue ‘Evolution and sustainability: gathering the strands for an Anthropocene synthesis’.

## 1. Introduction: the motivation for seeking to integrate evolutionary theory into social–ecological systems research

The rapid, human-induced changes in the Earth system during the Anthropocene [1] present our species with critical sustainability challenges, including the collapse of natural resources, the problem of climate change mitigation and adaptation, ecosystem degradation, and biodiversity loss [2]. Characterizing, disentangling and understanding these challenges and the processes that underlie them is a prerequisite for effective action. To that end, social–ecological systems (SES) research, a subfield of sustainability science, recognizes that humans and nature are deeply intertwined and aims to enable changes in governance, technology and behaviour that ensure a sustainable liveable planet for future generations [3,4]. SES research provides a useful toolkit of theories and methods for understanding how these systems work, and how we might aim them towards long-term health and resilience.

The concept of the Anthropocene invokes the idea of change, in terms of both changes in human societies, and the changes humans have caused in the natural world. Through changes in subsistence strategies, technologies and the socio-cultural mechanisms that govern human groups (i.e. norms and institutions) human societies have been able to extract more resources from the planet and live at increasingly higher population densities. During the Anthropocene, our species has begun to exert significant, ongoing impacts on the evolution of other organisms [5], including driving the sixth global mass extinction event in the history of life on Earth [6]. Understanding the causes and consequences of social and ecological change requires well-developed theories that help target appropriate explanatory factors, generalize across cases, and account for complex social–ecological causality. While it has been increasingly recognized that theory has an important role to play in sustainability research and its application to address to problems [7–10], theories of SES change have been elusive.

Formal evolutionary theory has been broadly and usefully applied as a dynamic theory of change of complex phenomena in biology and increasingly in the social sciences. However, it has been surprisingly little used to understand how SES change (but see [11,12]). In this paper, we argue that understanding the mechanisms that produce changes in SES will require some degree of evolutionary understanding at various levels. We should clarify that we are not seeking to develop a new evolutionary theory in this paper, nor perpetrate a takeover of SES and sustainability research by evolutionary science. Instead, our goal is to encourage greater exploration, development and application of evolutionary theory within SES research. While it is likely that all SES contain evolutionary aspects, it is far from clear which processes of SES change can be usefully characterized as evolutionary and which cannot. Clarity on this issue could lead to developing and refining theories to study these processes and their influence on SES change. As we will argue, there is unlikely to be only one way in which to link evolutionary theory with SES research and many different types of theoretical integrations may prove useful.

Our goal in this paper is to carefully explore the potential connections between evolutionary theory and SES research. In doing so, we aim to provide a field guide for practical collaboration across different disciplines. We first start by sketching out the distinct intellectual traditions of evolutionary theory and SES research and some of the key ways in which they differ. We then attempt to map some of the terminological and conceptual connections between these two fields of research. We show how the two fields sometimes (i) use similar terms to refer to different concepts, and (ii) share similar concepts that are masked by differences in terminology. We then illustrate a set of approaches aimed at achieving a tighter integration between these fields.

## 2. Research traditions

In seeking to connect SES research and evolutionary theory more closely, an important first step is to lay out a basic understanding of some key aspects of the work, approach and questions of each tradition. It is important to emphasize that both SES research and evolutionary theory are broad-based research fields with a wide variety of approaches and focal areas. As such, we are only able to provide thumbnail sketches of each tradition below.

### (a) Social–ecological systems research

SES research is a subfield of sustainability science that builds on a complexity perspective [13] and an understanding that humans are embedded in the biosphere, which they both influence and depend on [14]. In this tradition, SES research draws on diverse scientific fields and humanities disciplines with the aim of examining, and pursuing solutions-oriented research for, sustainability issues that have urgent implications for human and ecological well-being [15]. A significant portion of SES research is action-oriented, aiming not just to understand how a system functions but to act to improve the sustainability or resilience of the system. As such, SES research is typically considered a normative science that aims to enhance sustainability and engages in transdisciplinary processes to integrate the values, interests and knowledge of relevant stakeholders into the search for solutions. The urgency of today's sustainability problems have sometimes pushed theories and theorizing to the background; however, as the field is maturing the importance of theories for both explanation and action is increasingly recognized [10].

SES research typically views SES as complex adaptive systems [3,13,16,17]. Such systems are characterized by emergence, a process of self-organization among heterogeneous components that leads to novel system-level properties, structures, behaviours or capacities that cannot be understood as a straightforward ‘sum of their parts’ [16]. This view implies that SES are so complex that there is always uncertainty about the causal connections between different elements of the system, and how the system may respond to some form of alteration. Much research has focused on the implications of this uncertainty for governance, and highlighted the need for adaptive and transformative capacity to target more sustainable pathways. Less research has so far focused on understanding and theorizing complex causality that underlies processes of emergence. In its attempts to understand and explain emergent SES phenomena, SES research draws from a variety of frameworks, including adaptive management and governance [18,19], resilience [3], commons research and the SES framework [20], social–ecological transformations [21,22], and social–ecological action situations [23]. In SES research, theory is often developed to help describe and explain novel complex situations, and theories may be drawn from a diversity of sources depending on the situation at hand, and not necessarily connected to one single overarching or unified theoretical framework.

SES research that aims to understand the complex dynamics of SES is often place-based and descriptive, in order to incorporate context-dependent relations and processes that shape pathways towards sustainable or unsustainable outcomes. For example, long-term research in the Pamir Mountains of Tajikistan has studied the effects of development interventions on poverty and biocultural diversity [24]. Some of this work has modelled the idea of ‘poverty traps’ to study the dynamics that cause ‘undesirable’ situations of poverty and environmental degradation to persist [25]. An SES perspective can help reveal unexpected and unintended consequences of interventions through explicitly focusing on the interrelated feedbacks within and between social and ecological dynamics. For example, an improved wheat variety that was introduced to the Pamirs in the 1990s failed to produce higher yields (the seeds failed after 2 years, lacking agricultural inputs), but also had deleterious effects on cultural and linguistic diversity in the region and led to a transition from agriculturally biodiverse fields to an abandoned landscape [24].

### (b) Evolutionary theory

Evolutionary theory attempts to explain the diversity of life, and the processes by which that diversity arises. A broad definition of evolution can be given as change in the inherited characteristics of a population over time. This focus on changes in the population contrasts with the kind of developmental changes that individuals go through in their lifetime. The key feature that distinguishes a process as being evolutionary is that there needs to be some means by which variation in the traits that individuals possess is generated, and that this variation needs to be transmitted (or inherited) between individuals in a population [26]. For example, mutations can occur that change the genetic sequence (i.e. the genotype) of an individual organism. These mutations can then be inherited by the offspring of this organism, meaning that these individuals have genetic sequences that differ from the rest of the population. While mutation is the immediate cause of changes in genetic material, other processes shape how widespread these new genetic variants become in the population. For example, in small populations, certain individuals may leave behind more offspring than others by chance, which can lead to their genes becoming more common in the population, a process known as drift. Sometimes new mutations have important effects on the observable characteristics of an organism (i.e. the phenotype) that enable individuals to survive better and/or produce more offspring. In such cases, those variants will become more common in the population (i.e. they have a higher fitness, table 1). In natural populations, this process of natural selection is amplified as there is often competition over limited resources (i.e. not all the individuals that are produced can survive). There are many aspects of evolutionary theory and it is beyond the scope of the present paper to cover them all. Here, we focus on some of the evolutionary concepts that we think are most relevant for connecting to SES research: processes of change in organism–environment interactions, cultural evolution and multilevel selection.

**Table 1.** Select examples of shared terminology between Evolution and Social-Ecological Systems research that have differences in meaning.

evolution	social–ecological systems	reflection
**Adaptation**. The dynamic process that leads to a fit between organisms and their environment owing to differential survival and/or reproduction [27]. Can involve genetic or cultural change (e.g. [28]).	**Adaptation**. Incremental change in a social–ecological system to address a problem [29].	Both definitions refer to a functional match. However, there are differences in the entities to which the adaptation brings benefits (organism versus social–ecological system), and the processes involved (see [30] for a discussion of this in relation to climate adaptation).
**Niche**. An ecological space in which a species can survive, dependent on environmental parameters such as temperature, rainfall, insolation [31].	**Niche**. Social spaces shielded from mainstream market selection that serve to incubate innovation [32,33].	The core difference lies in the protective nature of the ‘niche’ in SES. Ecological niches are not thought to be fundamentally protective. Also, in SES, 'niche' is only used for microlevel environments, but for evolutionists 'niche' is used more widely (see [34]).
**Coevolution**. Typically, the process of reciprocal adaptation that occurs between two species or between different inheritance systems (e.g. gene–culture coevolution [28]).	**Coevolution**. Emphasis on intertwinedness of social and ecological systems, reflects the fact that they can mutually influence each other to generate novel outcomes [35–37].	Differences in the type of entities that co-evolve; coevolution in SES has been used in a more conceptual way, for example to describe the connections between cultural and biological diversity [38–40].
**Social learning** in animal behaviour and cultural evolution means learning from others [41,42].	**Social learning** in SES research occurs when learning arises through social interactions and becomes situated in wider social units or communities of practice (e.g. [43,44]).	An important but subtle difference because, while both refer to social processes, they are actually quite distinct (see [45]).
**Transition**. The original concept of ‘major evolutionary transitions’ [46] has been refined to ‘evolutionary transitions in individuality’ (ETI) which explain the emergence of higher levels of organization from lower level units (e.g. [47]).	**Transition**. Sustainability transitions [48] and transformations [21,22,49] represent a change within a socio-ecological system to a distinctly new state (whether that state is sustainable or not).	An evolutionary transition in individuality (ETI) is a specific mechanism which may or may not apply to social–ecological processes, and is a domain-specific system-level change. The term ‘sustainability transitions’ is often used in a normative sense to indicate that the aim of enabling a transition is to achieve sustainability.
**Traps.** An evolutionary trap is a situation in which a previously adaptive trait becomes maladaptive. For example, traits to exploit a resource that becomes unusable in the future [50].	**Traps.** Social–ecological traps are persistent, undesirable situations characterized by reinforcing feedbacks [51,52].	Both capture path dependency but evolutionary traps are focused on inherited traits and genetic constraints and social–ecological traps on system behaviours and states.
**Fitness,** or Darwinian fitness, in evolutionary biology, is the average reproductive success of a set of organisms of a certain type [53,54].	**Fit.** The matching of institutions to characteristics of the environment, such as its spatial and temporal heterogeneity [55].	Evolutionary fitness is a population-level measure, while institutional fit can be evaluated for a single human organization. Both are relevant in SES change.

Evolutionary thinking has been used to explain the reasons why organisms appear to be well-designed or well-matched to their environments in the absence of intentional design. Adaptation occurs when changes in the inherited traits of a population make organisms better able to live in their environment. In biological systems, adaptation occurs through natural selection, i.e. individuals that have phenotypic characteristics that fit better to the environment will be better able to survive and/or reproduce and therefore have a higher probability of leaving behind offspring that inherit the same characteristics. In humans, both genetic and cultural adaptations can occur. For example, Inuit people are able to live in the Arctic today owing to the clothing, buildings, and hunting practices and technologies that their ancestors developed and refined over generations. Furthermore, the high-fat diet of such populations has led to selection for genes that help desaturate fatty acids and distribute fat in the body [56]. Organisms can also modify their environments to better suit their existing traits and therefore enhance their survival, a process known as niche construction [57]. Humans have been prolific niche constructors in the Anthropocene and so generated novel ecosystems (i.e. farms, fisheries, cities). Agriculture is a particularly visible means by which populations have altered their environments to increase the amount of resources they can extract from them. Agriculture has also caused extinctions, changed species distributions (e.g. the global spreads of wheat, rice, cattle, dogs and rats), and altered abiotic environmental factors, sometimes with negative results that have led to problems later [58].

As we have touched upon above, the logic of evolutionary thinking can apply to non-genetic systems too. There are many researchers working on the implications this has to our understanding of how evolution works (broadly known as the extended evolutionary synthesis (EES) [59]. In humans, for example, information affecting behaviour is predominantly transmitted culturally through social learning. This results in cultural evolution in which behaviours, language, institutions and technology are modified and spread between people and groups [28,60]. Cultural evolutionary theory attempts to integrate an understanding of our evolved biological and psychological capacities, how and why individuals learn from others, and the population-level consequences for the spread or persistence of different behaviours and traits. Governance systems and organizations involve the establishment of institutional rules that affect how resource users behave, which themselves are innovated, altered, transmitted and selected, in an evolutionary process [61–63]. Cultural evolutionary theory not only provides a framework for understanding why our species has been so successful [64], but also offers insights into why sometimes people and societies appear to act in harmful or maladaptive ways [65]. This broader interpretation of evolution means that evolutionary theory can be applied to understand changes in SES that are not due to biological or genetic changes. Indeed, for understanding social change in SES, cultural evolutionary theory is likely to be most relevant. For example, it can be applied to understand how pro-environmental behaviours may be copied in a population, or how institutional rules that protect common-pool resources emerge and change.

Although evolutionary thinking as applied to human social and cultural systems is based on the fundamental concepts of variation, inheritance and competition, this does not mean that processes of cultural evolution are exactly the same as in genetic systems [66]. For example, inheritance in cultural evolution is based on social learning rather than being tied to biological reproduction or transmission of genetic material. Also, in contrast to genetic mutations, the generation of variation in cultural traits is not necessarily random. Indeed, humans are capable of forward-planning and making goal-directed changes to behaviours or social systems (i.e. there is guided variation) [67]. It is true that if people are able to solve adaptive problems relatively straightforwardly then there is little need to invoke evolutionary explanations. However, often the problems faced are complex or causally opaque, in which case attempted solutions may only be partial improvements. Under these circumstances, evolutionary thinking is still relevant. Solutions may need to build incrementally in a cumulative process over extended periods of time [68]. When different solutions are being attempted, then some may be better than others and therefore more likely to be copied or otherwise survive (i.e. some form of selection is occurring). In SES research, the uncertainty of knowing whether interventions will be effective or not has led to the approach of adaptive management, whereby interventions are introduced and then assessed and modified based on this feedback [69]. Recent models of institutional development show how evolutionary processes can be combined with some degree of foresight in individuals, enabling a group to find solutions to collective action problems [61,70]. Although guided variation can still be involved in evolutionary processes, one effect it can have, in contrast to random variation, is to speed up the rate at which adaptation occurs [71].

It is also useful to highlight that evolution and selection can occur at different levels of organization, both above and below the individual (i.e. multilevel selection). In hierarchically ordered organisms, how things evolve will depend on the balance of selective forces acting at different levels, and higher levels of biological function and integration emerge owing to selection at higher levels [72]. Multilevel selection has a somewhat controversial history, which is beyond the scope of this paper, and it is often argued that the conditions required for selection act on groups of individuals (i.e. group selection) do not commonly occur in biological systems ([73], but see [74,75]). In particular, migration or gene flow between groups can reduce the variation between those groups that is required in order for group selection to be a significant force. However, in cultural systems, processes such as conformity, or frequency-dependent copying, can maintain cultural distinctiveness between groups even when there is physical migration of individuals. Therefore, in human groups or social systems, cultural group selection is a plausible mechanism and may occur owing to the differential survival of groups (e.g. some groups outcompete or replace others), copying of group-level traits (e.g. the traits of successful groups are more likely to be borrowed), or biased migration (e.g. people move to more successful groups) [76]. SES often have different levels of organization and governance, and cultural group selection may be an important evolutionary mechanism for understanding how sustainable behaviours and institutions can evolve [12].

## 3. Navigating the intersection

As two fields of academic inquiry that seek to understand and explain connections between organisms and their environments, evolutionary theory and SES research share some common characteristics. The two fields share an interest in complex living systems, and a similar focus on the distinction between phenomena that endure and those that are more transient. Indeed, SES research has drawn on evolutionary concepts and terminology since its inception [77]. Both fields employ rich abstract and general theories that encompass a large set of cases, and both have also borrowed concepts and theoretical tools from other fields. For example, SES research has emerged by bringing together multiple strands from the natural and social sciences, while evolutionary theory has borrowed and developed theoretical frameworks from fields such as economics (e.g. [78]). Here, we attempt to demonstrate where evolutionary processes are likely to be taking place within SES, and highlight where specific areas of evolutionary theory may be of particular practical usefulness. We then go on to provide a demonstration of how mapping and identifying potential evolutionary processes in Anthropocene SES may be a practical way to aid integration of evolutionary thinking into SES research. To highlight the potential for an integration between the fields to help address some of the challenges of the Anthropocene, we finish this section by exploring how evolutionary thinking may be of use in better designing interventions for intentional beneficial change.

### (a) Navigating concepts and terminologies

To make connections between evolutionary theory and SES research in a way that leads to productive collaborations and meaningful insights, it is necessary to have a set of clearly defined core concepts and an understanding of the terminology used to describe these concepts. SES research already employs a set of evolutionary terms. For example, ‘adaptive capacity’ and ‘coevolution’ are common in social–ecological research. However, many such borrowed terms have different meanings and are used in different contexts. Some terminology was adopted in more of a metaphorical sense (e.g. 'coevolution' is often used metaphorically) while other terms were intended as more explicit theoretical links. However, creating more robust theoretical links between the fields requires a set of shared terms that are both detailed and precise. Thus far, this collaborative bedrock has been missing. Our discussions as a group of researchers from a variety of different disciplinary backgrounds, and attempts by some of us to navigate the intersections between these bodies of science, have revealed that the two fields often use different terms to refer to somewhat similar concepts (see below). Perhaps even more confusingly the two fields employ similar terms to mean different things. An illustrative and important example of the latter issue is the term ‘social learning’ [45]. In behavioural science and cultural evolution, ‘social learning’ is defined as learning through interactions with conspecifics or their products (e.g. [42,79–81]), and is distinguished from ‘individual learning’, which refers to learning that occurs in the absence of any influences from other individuals. By contrast, for SES, ‘social learning’ occurs when learning arises through social interactions in a group and becomes situated in wider social units or communities of practice [44].

Different definitions exist, and allowing them to collide without being aware of this can lead to confusion. In table 1, we identify several cases where the same (or very similar) terms are used to refer to different concepts in evolutionary science and SES research respectively. We do not suggest that table 1 (and table 2) is comprehensive, but rather it serves to illustrate that thought needs to be taken when attempting to bring in ideas from evolutionary theory. We hope that an awareness of such differences will help avoid potential misunderstandings and improve communication between researchers from both fields.

**Table 2.** Select examples of similar concepts in Evolution and Social-Ecological Systems research where different terminology is used.

evolution	social–ecological systems	reflection
**Evolvability** is defined as the capacity of a system or a population to change via adaptive evolution when required [82]. For example, genetic diversity can help a population survive novel environmental changes rather than go extinct.	**Resilience** is defined as the capacity for a system to persist in the face of changes and shocks. This can involve changes to the system, including adapting and reconfiguring [29]. Also: adaptability, transformability.	SES and evolution are closely aligned on this concept. In SES, a governmental system that is able to adapt to changing social or ecological conditions, or in biology where developmental systems are structured in ways that make adaptive changes more likely.
**Environment**, in evolution, refers to a broad, abstract concept of all biotic and abiotic factors with which an organism interacts.	**Context**, in SES research, refers to the particulars of the social, ecological, institutional or other factors that determine the unfolding of social–ecological events.	Both depict the influences that may shape the traits of individuals or systems.
**Evolutionary constraint**. Factors that make it difficult for certain changes in traits (particularly towards adaptive traits) to occur.	**Social–ecological rigidity trap.** Strong self-reinforcing controls preventing the flexibility needed for adaptation [83].	In both cases, there are factors that prevent individuals and/or systems from reaching states that would be more ‘beneficial’.
**Fitness landscapes or adaptive landscapes**. Originally, a visualization of the relationship between genetic or phenotypic state and fitness or reproductive success. Adaptive evolution occurs as populations move towards local optima which might inhibit the search for a global fitness optimum [84].	**Stability landscapes** is a way to conceptualize possible states of a system with regards to which states are most stable. Desirable states may not be particularly stable. Stability can be a property of the components of the system and may not relate directly to the functional performance of the system (e.g. [85]).	In both cases, locations on the landscape describe macro-state configurations, some of which may be easier to enter/achieve, and some of which may affect how easy it is to move to another configuration. In both cases, the landscapes may be dynamic rather than fixed.

It is also constructive to identify and clarify some shared concepts that are, nevertheless, described using different terminology (table 2). These ‘connections in meaning’ constitute a hidden conceptual alignment, and provide an opportunity to pull together shared frameworks that could lead to deeper understanding of evolutionary processes in SES. For example, ‘evolutionary constraints’ and ‘social–ecological rigidity traps’ (table 2) both impede a population or system in reaching beneficial or adaptive states. While evolutionary researchers more commonly explore genetic, physiological and ecological constraints, SES researchers commonly include social and institutional factors, often emphasizing self-reinforcing feedbacks. Therefore, connecting the two could be very instructive.

### (b) Navigating theoretical connections

The two research traditions have substantial empirical and theoretical connections. One important connection is game theory. Game theory has played a key role in the development of fields that SES draws from (e.g. economics, political science), and has also been critical in biology to understand how social systems emerge and change over time (i.e. social evolution). In biology, evolutionary game theory has been used to focus on how the strategies that individuals in a population employ change over time. Interestingly, the equilibria that are reached and which strategies ‘win out’ in the long run (i.e. are ‘evolutionarily stable’) can sometimes be different from analyses using classical game theory [86]. Evolutionary game theory may be valuable in understanding the dynamics of behaviour change and the factors affecting the adoption of sustainable behavioural norms. For example, a model by Lafuite *et al*. [87] shows how time-lags between the use of extractive agricultural technologies that cause biodiversity loss and the subsequent loss of ecosystem services that might result from biodiversity can inhibit the adoption of sustainable norms, leading to a population-overshoot-and-collapse crisis. Furthermore, a model by Schlüter *et al*. [88] explores how resource abundance, variability and connectivity may affect sustainable extraction of shared resources. They find that sustainable resource is seen under conditions of no-variability, but that this can be disrupted by increased abundance or small increases in resource variability. By contrast, sustainable use may be stabilized by resource scarcity or a large degree of variability. Additionally, if groups are connected ecologically but separate socially then there is the possibility that sustainable actions in one group could negatively impact another. These findings may have implications for understanding how climate change and geo-political relationships between countries may affect resource use and political stability. Another advantage of evolutionary game theory is that it is well designed to study the evolution of cooperation [89], which is intimately connected to understanding how solutions to collective action problems in natural resource management can be overcome [90,91]. Such an approach can examine the role of different ‘basins of attraction’ in catalysing cooperation [11] and on analysing the ensuing dynamics [92], which may have practical applications for designing sustainable management solutions to the challenges of the Anthropocene (see §4c ‘Designing interventions for intentional beneficial change’ below).

**Figure 1.**
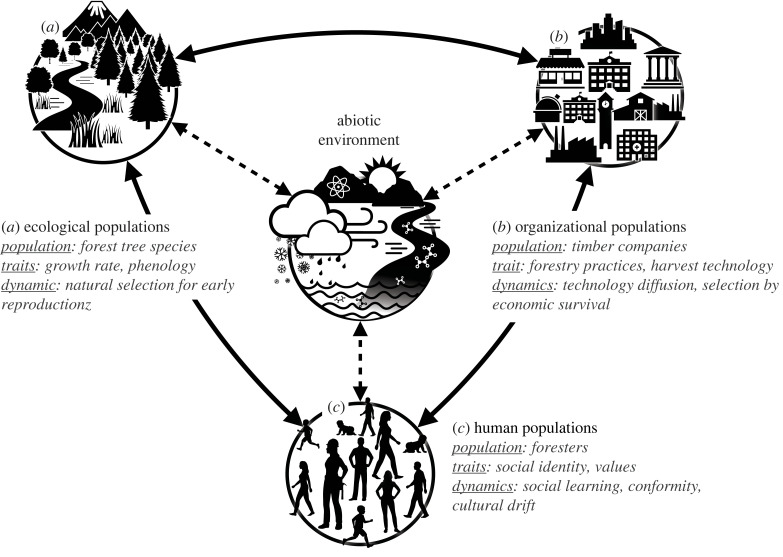
Social–ecological systems (SES) contain multiple components that are themselves evolving populations. A simple application of evolutionary thinking highlights that evolutionary processes can occur in different parts of a social–ecological system, including (*a*) trait evolution in wild or managed species, (*b*) institutional evolution in organizations, and (*c*) cultural evolution of human behaviour. An example of a forestry social–ecological system is presented to illustrate the kinds of populations, traits and dynamics involved in applying evolutionary thinking to a social–ecological system. Arrows represent the possible evolutionary connections between different evolving populations in the system as well as the abiotic environment.

As we have seen above, evolutionary theory has implications for thinking about both the ‘ecological’ and the ‘social’ components of SES. In figure 1, we present a modified version of the kind of diagram that is commonly used in SES research [20,93,94] to illustrate where theoretical connections may be most readily made. Biological populations (figure 1*a*), which can include the resources that humans use, may experience evolutionary change such as selection or extinction, while eco-evolutionary dynamics describe the mutual feedback between ecological change (environment) and evolutionary change of the population. An example of such dynamics is density-dependent selection, where the relative performance of individuals changes with the density of the population. The social aspects of figure 1 identify human populations (figure 1*b*), and ‘populations’ of organizations (figure 1*c*), which involve the governance structures and institutional rules that shape individual behaviour. Evolutionary theory is being increasingly applied to understand human behaviour and how the diversity of cultures and societies has arisen [71]. There are many ways in which evolutionary thinking can be applied to understand changes in human and organizational populations, including for example: (i) how the history of biological and cognitive evolution in our species continues to shape the ways people in contemporary societies behave, (ii) how social learning rules affect the spread of behaviours, (iii) how cooperation and collective action in humans evolve (including the role of norms and institutions), or (iv) how cultural diversity at different levels changes over time. At the centre of figure 1, we have the abiotic environmental factors (e.g. climate, terrain, water) that can affect the evolving populations. Change in abiotic factors can occur through physical processes (e.g. changes in solar activity, tectonics). Such changes are not considered to be evolutionary in the sense we have been discussing change in this paper. However, they can also change as a result of their interactions with the evolving populations [95].

As well as applying evolutionary thinking within these nodes on the diagram, we can also consider evolutionary interactions between the nodes (as represented by the arrows). For example, evolutionary change in biological populations can be driven by human influence as well as natural processes. In conservation science, an understanding of genetic diversity and how populations can adapt to changing conditions is essential for formulating strategies to mitigate the impacts of climate change or other anthropogenic disturbances to ecosystems. Undesirable invasive species may sometimes be adapting too quickly, while desirable species may be adapting too slowly (i.e. creating an evolutionary mismatch [96] between organisms and their environment). Ecological populations and abiotic factors may provide ecosystem services, and, while challenging, it is possible for the social systems to shape environments and modify selection pressures intentionally for long-term human benefit and ecological stability [93,94,96]. For example, the global (mis)use of antibiotics has led to the evolution of microbial resistance to antibiotics, which threatens global health infrastructure. Therefore, to preserve antibiotic medicines we must manage microbial evolution [97]. Ecological populations and abiotic factors can also shape social systems, and fields such as human behavioural ecology investigate the evolutionary processes that enable human behaviours and systems of organization to be well adapted for certain ecological contexts [98]. Furthermore, humans affect their environment through cultural niche construction, with those changes being carried forward (ecological inheritance) to affect human populations and organizations in the future [99]. These perspectives clearly align with social–ecological coevolution [39,40] (table 1), but apply at different levels of generality and emphasize different processes (e.g. [100]). While initial work has explored similarities and differences between these approaches, the literatures are still fairly isolated and this is a potentially fertile research frontier.

Organizations and individuals represent nested layers in social systems that can interact with each other and with ecological systems in many ways. For example, the cultural–historical background of different populations may help in understanding the dynamics of spread of institutions [61,101,102], which could be of value in understanding how sustainability projects or conservation schemes can be successfully implemented in different places. Research on socio-technical transitions has developed a framework that integrates evolutionary theory to understand how technological innovations spread and become dominant in societies [103]. Geels' [104,105] framework has been influential in research on SES transformations, where it was merged with a framework that emphasizes different phases of (governance) transformations [21,22,106]. An approach that is different in multiple regards is that of Waring and collaborators, who propose a more mechanistic model of multilevel selection on cooperative environmental behaviours [12]. Waring and co-authors propose that cultural group selection is an evolutionary process that may, under the right circumstances, lead to the evolution of sustainable behaviours and institutions. Specifically, sustainable social systems evolve when the strength of cultural evolutionary selection is greater on groups for environmental conservation than it is on individuals for environmental consumption [12].

## 4. Working at the intersection

In the previous section, we saw how there are multiple ways in which evolutionary theory may be usefully integrated into SES research. Researchers interested in exploring these connections further will need to decide which processes are most relevant for a particular research question or study system. In this section, we provide some specific, practical guidance about how useful insights at this intersection may be achieved. We start by first laying out, in general terms, a conceptual tool for systems mapping exercises for identifying where evolutionary processes may be most usefully connected to SES research and challenges (§4a) [107]. In §4b, we explore how aspects of theory in developmental evolutionary biology may serve as a template to examine change and evolution in SES. Finally, we provide an example of how evolutionary theory may be used in a more applied way to help design interventions for intentional beneficial change.

### (a) Mapping evolutionary processes in Anthropocene social–ecological systems

Systems mapping is a common approach with which SES researchers start exploring complex problems by diagramming system components, processes and relationships [108]. When combined to include mapping of evolutionary processes, for SES researchers, this can help appreciate and articulate the many specific evolutionary processes through which SES evolve. For evolutionary researchers, it can provide an avenue for starting to think of subsets of questions that can be addressed with existing models or to identify requirements of new modelling approaches that deal with the complexity of a particular social–ecological system.

**Figure 2.**
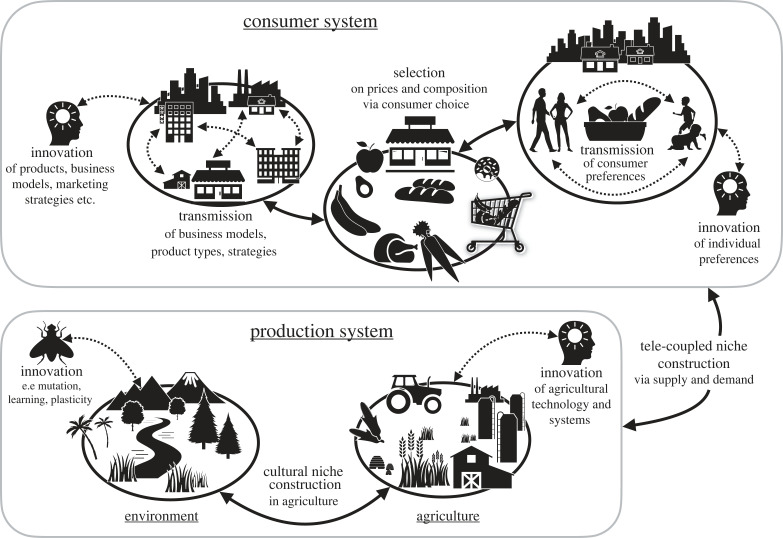
Mapping an Anthropocene food system. Numerous evolutionary component processes may be identified in the interlinked consumer–industry–social–ecological systems of the Anthropocene (see text for more details).

Figure 2 illustrates the mapping of innovation (mutation), transmission (`inheritance’), selection and niche construction processes in an archetypical Anthropocene food system where locations of production and consumption are separated in space and connected via global supply chains (e.g. [109–34]). In this example, the goal is to understand the processes that shape the spread of sustainable and healthy diets, a major leverage point for achieving environmental sustainability and basic outcomes of health for all, as identified in the sustainable development goals [111]. In the consumption system, examples of transmission and innovation processes are seen in how consumers transfer and modify social norms around preferences for sustainable and healthy diets (e.g. [112]) and how businesses may get inspired by each other in how to promote sustainable and healthy diets. Based on their preferences, consumer demand imposes one of multiple selection pressures acting on the composition and pricing of sets of commodities [107]. In a form of tele-coupled niche construction, these dynamics in the consumer system influence incentives for certain production practices and choices in a distant production system (which also affects local niche construction processes in another country), and the supply of goods from the production system feeds back to influence cultural evolutionary dynamics in the consumption system (see e.g. [34,113]).

This mapping helps achieve two things: (i) it makes the seemingly complex and abstract dynamics more concrete by identifying fundamental evolutionary processes that interact to produce overall dynamics, (ii) it identifies candidate processes for integrated evolutionary SES modelling of a social–ecological system (in this case food systems). As part of such a mapping exercise, some evolutionary concepts might be found more valuable than others for SES researchers. This understanding might occur from just the mapping exercise itself, or it may come later when, in a full system dynamics model, these relationships would be turned into linked sets of equations.

### (b) Understanding the evolution of systems: taking inspiration from evo–devo research

Another strand of evolutionary theory that may be of particular value in examining change in SES comes from developmental evolutionary biology (evo–devo). Evo–devo theory emphasizes the role of developmental mechanisms in the origin of phenotypic novelty and evolutionary change, from the evolution of new morphologies to the evolution of behaviour [114]. In other words, rather than treating the generation of variation as a ‘black-box’, as often happens in fields such as population genetics or evolutionary ecology, evo–devo examines how new phenotypes can emerge owing to changes in the systems that affect how organisms develop during their lifetimes. This approach can provide insights into whether certain phenotypic changes are more or less likely than others owing to the way that systems are structured or organized. This approach has also revealed that differences in phenotype within or between populations may not be the result of different genotypic differences but rather can result from *developmental plasticity*, the capacity to develop in different but functionally appropriate ways in response to the environment [115]. Understanding and being able to distinguish which changes in SES are due to population processes and which are due to changes in system inputs may prove extremely useful itself. Concepts such as modularity, evolvability and the importance of developmental constraints are used in evo–devo to make sense of the interrelations between systems of organization and evolutionary change. Many of these have clear relevance for the historically contingent and modular complex systems that often constitute elements in SES.

One compelling example is the evolution of technology, which is likely to be a crucial aspect of any human transition to sustainability. Here, a general modular structure (e.g. [116]), together with constraints, historical contingency, and the concept of ‘evolvability’, have been proposed [117]. In attempting to understand and describe general principles for the evolution of technology, Arthur [116] proposed that underlying numerous different technologies is a common modular structure. In evo–devo, ‘modules’ are defined by relatively high internal integration and relatively low external separation, their persistence through time, their reuse across different species (or in this case, technologies), and the possibility for their autonomous response to selection [118]. If technologies are typically composed of identifiable and persistent modules and respond to selection in this way, then evo–devo thinking may play a crucial role in facilitating our understanding of the complex and interacting evolutionary processes involved in SES. Kauffman *et al*. [117] have examined how ‘evolvability’ (a system's ability to change over time [119]), together with the role of constraints on change due to historical circumstances, can be examined in a ‘fitness landscape’ model of the evolution of technology. Here, the historical path of a technology across a fitness landscape determined which parts of the relevant landscape could be most easily reached, and which innovative strategies were most economical and successful. Insights like these have the potential to increase the speed and effectiveness of technological innovation and to point to aspects of technology, like features of relevant fitness landscapes, that are useful targets for further research relevant to SES.

### (c) Designing interventions for intentional beneficial change

As well as achieving an enhanced abstract understanding of how SES change, evolutionary concepts can be used to specify potential interventions aimed at inducing intentional positive social change [120], which could potentially complement current approaches in adaptive management [121]. This work has a variety of flavours, from research designed to understand how SES might evolve to more sustainable states [91], to identification of cultural evolutionary tipping points that might influence the design of interventions [122], to applied collaborations using evolutionary concepts to rationalize and extend existing effective strategies [123,124]. For example, Andrews & Borgerhoff Mulder [125] identify where and how interventions offering payments for ecosystems services might shape the emergence and spread of cooperation over sustainable forest management practices. Hébert-Dufresne *et al*. [126] explore the wider conditions necessary for the spread of beneficial behaviour and institutions in group-structured populations. Wilson [127] advocates establishing coalitions of scientists, clinicians and consultants to guide cultural evolution through a more intentional, inclusive, and participatory social process, as seen in the ProSocial movement [128,129].

One specific and promising normative application of the ideas discussed in this paper entails deliberately facilitating positive social tipping, i.e. designing and deploying interventions that accelerate rapid and self-reinforcing transitions from entrenched unsustainable social norms to more sustainable alternatives [130]. A nascent literature emphasizes the large potential for carefully crafted target interventions, such as social information provision and behavioural nudges, to instigate self-propagating change from early adopters of the green behaviour or technology to the less enthusiastic later movers ([112,131,132], but see Maier *et al*. [133], Mertens *et al*. [134] and Milkoreit [135] for debates about the evidence for social tipping points and the effectiveness of behavioural nudges). To the extent that these interventions involve individuals learning or being influenced by what they perceive as the behaviour of others (rather than just responding independently to non-social environmental cues), then social learning processes and cultural evolutionary dynamics are likely to be in effect.

Furthermore, classical equilibrium stability analysis from economic theory [136], which assumes homogeneous preferences, has a hard time explaining how rare behaviours can come to dominate a population. Yet, in the real world, previously stable social conventions such as fertility norms, expectations about gender roles in the workplace and tolerance for smoking in public spaces can get quickly overturned by the efforts of committed minorities [137]. Understanding how such changes can come about if we have theories that allow individuals to be heterogeneous about the threshold level of adoption (population share) above which they are willing to change behaviour. This is something that can be accommodated by theories that emphasize off-equilibrium behaviour, as common in evolutionary game theory [138]. Once the tipping point is reached within a complex system, and a critical transition can occur, the actions of a minority group trigger a cascade of behaviour change that rapidly increases the acceptance of a minority view [139–142]. Notably, such behavioural cascades can be triggered by intentional infrastructural redesign, as with rapid evolution of the cycling culture in Amsterdam once bicycle lanes were installed, which enabled a critical number of individuals to start cycling more frequently and resulted in widespread imitation [122]. While most of the work on social tipping interventions has so far been experimental [137,143,144], deploying cultural evolutionary thinking within SES may help to illuminate the enabling conditions for social–ecological system tipping.

## 5. Conclusion

SES and evolutionary theory are potentially highly complementary conceptual frameworks, but integrating them effectively will require understanding the distinct research traditions of the different fields, and the specific terminologies and definitions that are employed to articulate key concepts. In this paper, we have drawn a distinction between the kind of population thinking that is commonly employed in evolutionary studies and the kind of systems thinking that is more prominent in SES research. Researchers seeking to work at the intersection of these fields will need to familiarize themselves with each as both views will apply in different aspects of SES change. Identifying which framework or combinations thereof provides a better summary of what particular SES processes should be a central goal of further research. Fields such as evolutionary developmental biology that integrate these different modes of change may provide a useful template for integration.

In this paper, we have argued that useful synthesis will identify the specific role that components of evolutionary thinking can contribute to the broader multidisciplinary tent of SES research. In particular, evolutionary approaches to human behaviour such as cultural evolutionary theory [28,60], human behavioural ecology [98], gene–culture coevolutionary theory [145,146] and cultural niche construction theory [147,148] offer perspectives and theoretical tools that could enrich existing approaches to human behaviour within SES research to help account for the complex, intertwined, context-dependent and emergent nature of many SES phenomena. As we saw above, a potentially useful aspect of such approaches is that they can help understand why human behaviour may deviate from that predicted under models of people as economically rational actors that often inform much work in the social sciences and public policy.

Our experience leads us to think that concrete and case-driven approaches to integration are more likely to be successful than a more general or abstract approach. We have attempted to illustrate this through a discussion of techniques such as systems mapping, or the identification of specific case studies. One way that integration can be accelerated is through collaborative modelling of a social–ecological system, which encourages the teams to determine how and why certain parts of a social–ecological system might evolve [9]. For example, rather than developing a new ‘grand theory’ [149], middle-range theories (e.g. [150]) which are context-based generalizations that apply to specific subsets of cases may be more attainable. A middle-range evolutionary SES theory would develop connections between a specific sustainability phenomenon, such as poverty traps or successful collective action in irrigation, and related evolutionary mechanisms, such as the evolution of cooperation or cultural evolution. Working in this way may also better address specific Anthropocene challenges such as improving the resilience or robustness of certain types of SES.

As we proceed with our ambition of integrating evolutionary theory into SES research we may find that evolutionary thinking is more useful for some issues than others, or that existing theories need to be modified or refined in some way. For example, the complexity of SES phenomena makes it difficult to focus on only one trait owing its embeddedness and interactions with many other traits. This is not a new challenge; indeed, most of the current evolutionary theories discussed in this paper have been developed by building on and extending prior theories. Moreover, theoretical connections between SES and evolutionary research will not necessarily flow in only one direction, and SES research can help shape and further extend evolutionary theory. For example, models of cultural evolution have generally focused on the spread of traits between individuals and have not incorporated explicitly how institutions work, or how institutional evolution may occur. Researchers seeking to develop models of institutional evolution (e.g. [61,151]) have been able to draw on the insights of key aspects of SES research such as the work of Ostrom [152] (see also [153]). The next step in our research programme will be to develop explicit models that combine SES approaches with evolutionary dynamics in order to illustrate the potential added value of evolutionary thinking.

The challenges of the Anthropocene force us to address major sustainability issues within very short timescales (i.e. the lifetime of individuals living today). Understanding and guiding complex social–ecological change require working across traditional academic disciplines to conduct more problem-oriented research. Such disciplinary integration is already underway in SES research. We know from prior work that evolutionary processes are at work in both social systems and ecological systems, and in the interactions between them, and that this can sometimes lead to changes over comparatively short timescales. We have therefore argued here that integrating evolutionary theory with SES research will be an important part of this effort, and can enable a better understanding of how and why variation is generated, and how it spreads or persists. Through such endeavours we feel that effective policies may be enacted that encourage positive change through both systems and evolutionary pathways.

## Data Availability

This article has no additional data.
